# Epidermal growth factor receptor status and Notch inhibition in non-small cell lung cancer cells

**DOI:** 10.1186/s12929-015-0196-1

**Published:** 2015-10-24

**Authors:** Efstathia Giannopoulou, Achilleas Nikolakopoulos, Dimitra Kotsirilou, Angeliki Lampropoulou, Sofia Raftopoulou, Evangelia Papadimitriou, Achilleas D. Theocharis, Thomas Makatsoris, Konstantinos Fasseas, Haralabos P. Kalofonos

**Affiliations:** Clinical Oncology Laboratory, Division of Oncology, Department of Medicine, University of Patras, Patras Medical School, Rio, 26504 Greece; Laboratory of Molecular Pharmacology, Department of Pharmacy, School of Health Science, University of Patras, Rio, 26504 Greece; Laboratory of Biochemistry, Department of Chemistry, University of Patras, Rio, 26504 Greece; Electron Microscopy Laboratory, Faculty of Crop Production, Agricultural University of Athens, Iera Odos 75, Athens, 11855 Greece

**Keywords:** Notch intracellular domain, Epidermal growth factor receptor, Non-small cell lung cancer cells, Apoptosis, Autophagy, Cell cycle arrest

## Abstract

**Background:**

Notch may behave as an oncogene or a tumor suppressor gene in lung cancer cells. Notch receptor undergoes cleavage by enzymes, including γ-secretase, generating the active Notch intracellular domain (NICD). The aim of the present study was to investigate the effect of DAPT, a γ-secretase inhibitor, in non-small cell lung cancer (NSCLC) cells, as well as the impact of epidermal growth factor (EGF) that is over-expressed by NSCLC cells, on Notch signaling. H23, A549, H661 and HCC827 human NSCLC cell lines were used, expressing various NICD and EGF receptor (EGFR) protein levels.

**Results:**

DAPT decreased the number of H661 cells in a concentration-dependent manner, while it had a small effect on H23 and A549 cells and no effect on HCC827 cells that carry mutated EGFR. Notch inhibition did not affect the stimulatory effect of EGF on cell proliferation, while EGF prevented DAPT-induced NICD decrease in H23 and H661 cells. The type of cell death induced by DAPT seems to depend on the cell type.

**Conclusions:**

Our data indicate that inhibition of Notch cleavage may not affect cell number in the presence of EGFR mutations and that EGFR may affect Notch signalling suggesting that a dual inhibition of these pathways might be promising in NSCLC.

**Electronic supplementary material:**

The online version of this article (doi:10.1186/s12929-015-0196-1) contains supplementary material, which is available to authorized users.

## Background

Lung cancer is the leading cause of cancer death. Its incidence and mortality is 52.5/100,000 and 48.7/100,000 per year, respectively [1]. Non-small cell lung cancer (NSCLC) is the most common subtype, accounting for approximately 90 % of lung cancer cases [1]. Most patients are presented with advanced stage disease and since patient outcomes are largely dependent on stage, 5 year survival rate for lung cancer remains low, at about 15 % [2]. Until recently, chemotherapy with cytotoxic agents was the only available treatment for lung cancer [1]. Advances in the research field have led to the elucidation of unique pathogenic mechanisms, critical pathways and molecules involved in cancer development, which have resulted in the introduction of targeted therapies as new treatment options in cancer. A typical example is the epidermal growth factor receptor (EGFR) activity that has been found to be overexpressed in about 50–80 % of NSCLC [3]. Anti-angiogenic agent bevacizumab and EGFR tyrosine kinase inhibitors erlotinib and gefitinib have been approved for NSCLC treatment [4, 5]. Moreover, continuous ongoing clinical trials investigate the efficacy and safety of anti-angiogenic and anti-EGFR agents combinations, as well as other agents such as multi-targeted anti-angiogenic tyrosine kinase inhibitors and anti-HER2 agents [6–8].

Despite the existence of many therapeutic options, the prognosis of patients with metastatic disease remains poor, with a median survival of about 12 months with the newer regimens. This shows how important the development of new strategies remains. Although targeted therapies are a step forward, it is essential to clarify the biology of lung cancer cells [9].

Notch is a transmembrane heterodimeric receptor with four distinct members (Notch1 to Notch4) in humans and rodents. Notch signaling pathway is initiated upon ligand binding, where the receptor subjects into two proteolytic cleavages. The γ-secretase, a complex enzyme, regulates the second cleavage of Notch, through which the Notch intracellular domain (NICD) is liberated to the cytoplasm and then enters the nucleus in order to activate the transcription of Notch targeted genes [10]. Notch is known for playing a key role in embryogenesis and organogenesis by regulating cell proliferation and differentiation [11]. In addition, Notch seems to be involved in carcinogenesis and a cell-type dependent profile has been observed with Notch to act as oncogene or tumor suppressor gene [12–14]. Both behaviors of Notch have been described in lung cancer. Notch 1 and 2 proteins are frequently expressed in NSCLC. Notch 1 is rarely expressed in small cell lung cancer (SCLC), whereas a subset of SCLC exhibit Notch 2 expression. It is suspected that Notch has a growth promoting function in NSCLC, whereas in SCLC it exerts an inhibitory effect [11, 12]. Research also shows potential cross talk between Notch pathway and others, such as EGFR and Wnt [15–19]. Taking those findings together, Notch pathway is currently under investigation, with various agents being tested as potential new therapeutic options for patients with NSCLC [20].

The cross-talk between the Notch and EGFR signaling has been previously described in genetic model systems, where these pathways can function in either an antagonistic or synergistic fashion, depending on tissue and developmental context [21]. However, the mechanism through which this interplay occurs, remains unknown [19]. In breast cancer cells, Notch pathway is used by cancer cells to compensate for EGFR targeted inhibition [22]. The case of breast cancer along with previous data from lung cancers cells demonstrating that Notch 1 might have a role in acquired resistance to gefitinib [23], prompted us to investigate the inhibition of Notch in NSCLC cell lines after cell treatment with γ-secretase inhibitor; N- [N- (3, 5- difluorophenacetyl)- 1- alanyl]- S- phenylglycine t- butyl ester (DAPT), as well as the impact of epidermal growth factor (EGF) in Notch signaling. Our data indicated that Notch inhibition was not effective in all NSCLC cells and this effect was dependent on EGFR protein levels and mutations.

## Methods

### Cell culture and reagents

NSCLC cell lines H23, A549, H661 and HCC827 were purchased from the American Type Culture Collection (ATCC) and cultured as manufacturer recommends. According to ATCC, H23, A549 and H661 cells express wild type EGFR, while HCC827 cells express mutated EGFR bearing E746-A750 deletion (https://www.lgcstandards-atcc.org/) as shown in Additional file 1: Table S1. Cells were cultured in RPMI 1640 medium with 2 mM L-glutamine and supplemented with 1 mM sodium pyruvate, 4.5 g/L glucose, 1.5 g/L sodium bicarbonate, 100 μg/ml penicillin G/streptomycin, 2.5 μg/ml amphotericin B, 50 μg/ml gentamycin and 10 % foetal bovine serum (FBS). Cells were cultured at 37 °C, 5 % CO_2_ and 100 % humidity.

DAPT (N- [N- (3, 5- difluorophenacetyl)- 1- alanyl]- S- phenylglycine t- butyl ester (DAPT, LY374973) and EGF were purchased from Sigma (Sigma-Aldrich Chemie GmbH, Germany). All experiments were performed according to the following conditions: After reaching 80 % confluence, serum starvation followed for 24 h. Cells were then treated with DAPT at the concentrations of 0.01, 0.1, 1, 10 and 20 μM or/and EGF at the concentration of 1 μg/ml. In case of DAPT and EGF co-treatment, EGF was added 30 min prior to DAPT. The duration of the treatment was determined by the assay that followed.

### Cell proliferation assay

To determine whether DAPT alone or in combination with EGF, affects the proliferation of cell proliferation, the 3-[4,5-dimethylthiazol-2-yl]-2,5-dimethyltetrazolium bromide (MTT) assay was used, as previously described [24]. Briefly, cells were seeded at a density of 1,5 ×10^4^ cells/well in 24-well plates and cells treated as described above. Forty-eight hours after agents’ application, MTT solution (5 mg/ml in PBS) was added at a volume equal to 1/10, to each well and incubated for 2 h, at 37 °C. Medium was removed and 100 μl acidified isopropanol (0.33 ml HCl in 100 ml isopropanol) was added in each well in order to solubilize the dark blue formazan crystals. The solution was transferred to 96-well plates and was immediately read in a microplate reader (Tecan, Sunrise, Magellan 2) at a wavelength of 570 nm using reference wavelength 620 nm.

### Apoptosis assay

All cell lines were plated at 1 × 10^5^ cells per well in 6-well plates. DAPT was added as previously described. At the end of a 24 h incubation, cells were washed twice with PBS, trypsinized for 7 min and centrifuged for 4 min at 166 g. Cells were resuspended in 200 μl 1X binding buffer (10 mM HEPES pH 7.4, 140 mM NaCl, 2.5 mM CaCl_2_). The cell suspension was incubated with 5 μl Annexin V-FITC in the dark at 25 °C, for 10 min. Then, 10 μl of the 20 μg/ml propidium iodide stock solution was added, followed by 200 μl of binding buffer and the cells were immediately analysed by flow cytometry [25] (EPICS-XL of Coulter) according to the manufacturer’s instructions (rh Annexin V/FITC kit, Bender MedSystems, Germany). The application of Annexin-V (An) along with propidium iodide (PI) distinguishes 4 populations; the viable (An^−^/PI^−^), the early apoptotic (An^+^/PI^−^), the late apoptotic (An^+^/PI^+^) and the necrotic (An^−^/PI^+^) cells. The sum of early and late apoptotic cells calculated as apoptotic cells.

### Cell cycle analysis

All cell lines were plated at 1 × 10^6^ cells per Petri dish and DAPT was added as earlier described. At the end of the 24 or 48 h incubation, cells were washed twice with PBS, trypsinized for 7 min and centrifuged for 4 min at 166 g. Cell cycle analysis was performed using the Muse™ Cell Cycle kit according to the manufacturer’s instruction (Merck-Millipore, Germany). Briefly, cells were washed once with PBS and centrifuged for 5 min at 300 g. The supernatants were discarded leaving approximately 50 μl of PBS. The pellet of cells was resuspended in the residual PBS was added drop-wise into a tube containing 1 ml of ice cold 70 % ethanol while vortexing at medium speed. The samples were kept at −20 °C for at least 3 h. Then, 200 μl of fixed cells were centrifuged at 300 g, for 5 min. The pellet of cells was resuspended in 200 μl of Muse™ Cell Cycle Reagent and cells were incubated for 30 min, protected from light. After the incubation, cells were analysed by Muse™ Cell Analyzer, according to the manufacturer’s instructions (Muse™ software, Merck-Millipore, Germany).

### Western blot analysis

Cells were plated at Petri dishes. After reaching 80 % confluence, cells were treated as described above. At the indicated time point after agents’ application, cells were collected with scrapper and lysed using lysis buffer (50 mM Tris–HCl pH 7.5, 150 mM NaCl, 5 mM EDTA, 1 % Triton, 10 % glycerol, 1 mM phenylmethyl-sulphonyl-fluoride, 2 mM Na-orthovanadate and 10 mM leupeptin). Protein concentration was determined by Bradford assay. Samples were analysed by immunoblotting as previously noted [24]. For Beclin-1, an autophagy indicator, cells were collected 24 h after appropriate treatment and a goat anti-Beclin-1 (dilution 1:500, Santa Cruz, CA, USA) was used. For Notch-1 intracelluler domain (NICD), time course experiments were performed and a sheep anti-Notch-1 ICD (dilution 1:1000, R&D Systems, Germany) was used. For EGFR detection, time course experiments were performed and a rabbit anti-EGFR (dilution 1:6000, Millipore,Temecula, CA, USA) was used. Actin was used as internal control for protein quantification and a monoclonal anti-actin antibody (dilution 1:1000, Chemicon, Millipore, Temecula, CA, USA) was used.

Detection of the immunoreactive proteins was performed by chemiluminescence using horseradish peroxidase substrate SuperSignal (Pierce, Rockford, IIL, USA), according to the manufacturer’s instructions.

### Transmission electron microscopy (TEM)

H661 cells were plated at 1 × 10^5^ cells per well in 6-well plates. DAPT was added as previously described. At the end of a 24 h incubation cells were washed with PBS once, fixed with 2.5 % glutaraldehyde at 4 °C for 2 h and then washed twice with PBS. Cells were dehydrated in a graded series of ethanol (50, 70, 80, 90 and 100 %), transferred in to 100 % acetone for 15 min and embedded in SPURR resin. Ultrathin sections were stained with uranyl acetate and lead citrate and examined and photographed with a JEOL 100S transmission electron microscope equipped with an Olympus MegaView G2 digital camera.

### Statistical analysis

Differences between groups and controls were tested by one-way ANOVA. Each experiment included at least triplicate measurements. All results are expressed as mean ± SEM from at least three independent experiments.

## Results

### Profile of NSCLC cells according to NICD and EGFR protein levels

**Fig. 1:**
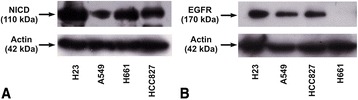
Screening of NSCLC cell lines for **a**) NICD and **b**) EGFR protein levels. The images of western blot analysis are representative of three independent experiments

Initially, NSCLC cell lines H23, A549, H661 and HCC827 (Fig. 1a and b) were screened for NICD and EGFR protein levels. Among four cell lines, H23 expressed the highest, H661 and HCC827 intermediate and A549 the lowest NICD protein levels (Fig. 1a). Concerning EGFR, H23 expressed the highest, A549 and HCC827 intermediate and H661 the lowest, almost undetectable, protein levels (Fig. 1b). According to ATCC, H23, A549 and H661 cells have wild type (wt) EGFR and HCC827 cells have mutated (mt) EGFR (https://www.lgcstandards-atcc.org/) (see Additional file 1: Table S1).

### The effect of Notch inhibition in NSCLC cells’ proliferation

**Fig. 2:**
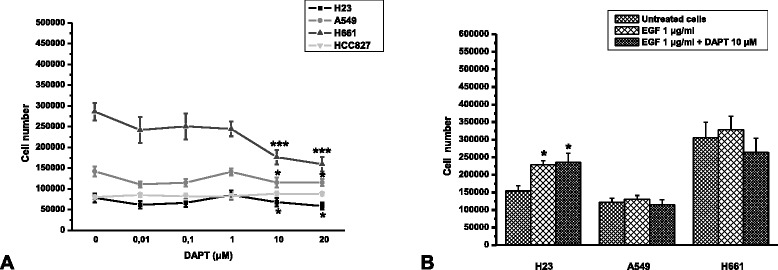
The effect of DAPT **a**) alone and **b**) after stimulation of NSCLC cells with EGF in cell proliferation, 48 h after DAPT application. Each value represents the means ± SEM from at least three independent experiments. The asterisks denote statistically significant difference between experimental groups and untreated cells. C *P < 0,05 and ***P < 0,0001

Since all cell lines express activated Notch, we studied the effect of DAPT on the number of unstimulated cells. The number of H661 cells was significantly decreased in a concentration-dependent manner 48 h after DAPT addition. DAPT had only a minor effect on H23 and A549 cells, while HCC827 cells were resistant to DAPT at any tested concentration (Fig. 2a). Based on these data, the inhibitory effect of DAPT on the number of cells initiated at 10 μM and thus this concentration was used for the following experiments.

To investigate a possible implication of Notch pathway with EGFR signalling, we stimulated cells with EGF 1 μg/ml for 30 min prior to addition of DAPT. HCC827 cells were not used in these assays, since they carry active mutated EGFR (see Additional file 1: Table S1) and do not further respond to EGF [26, 27]. EGF alone significantly increased only the number of H23 cells, while it had no effect on A549 and H661 cells. DAPT did not reverse the stimulatory effect of EGF in H23 cells. On the other hand, in all cells, the presence of EGF prevented the inhibitory effect of DAPT (Fig. 2a and b).

### The effect of DAPT in NICD and EGFR protein levels

**Fig. 3:**
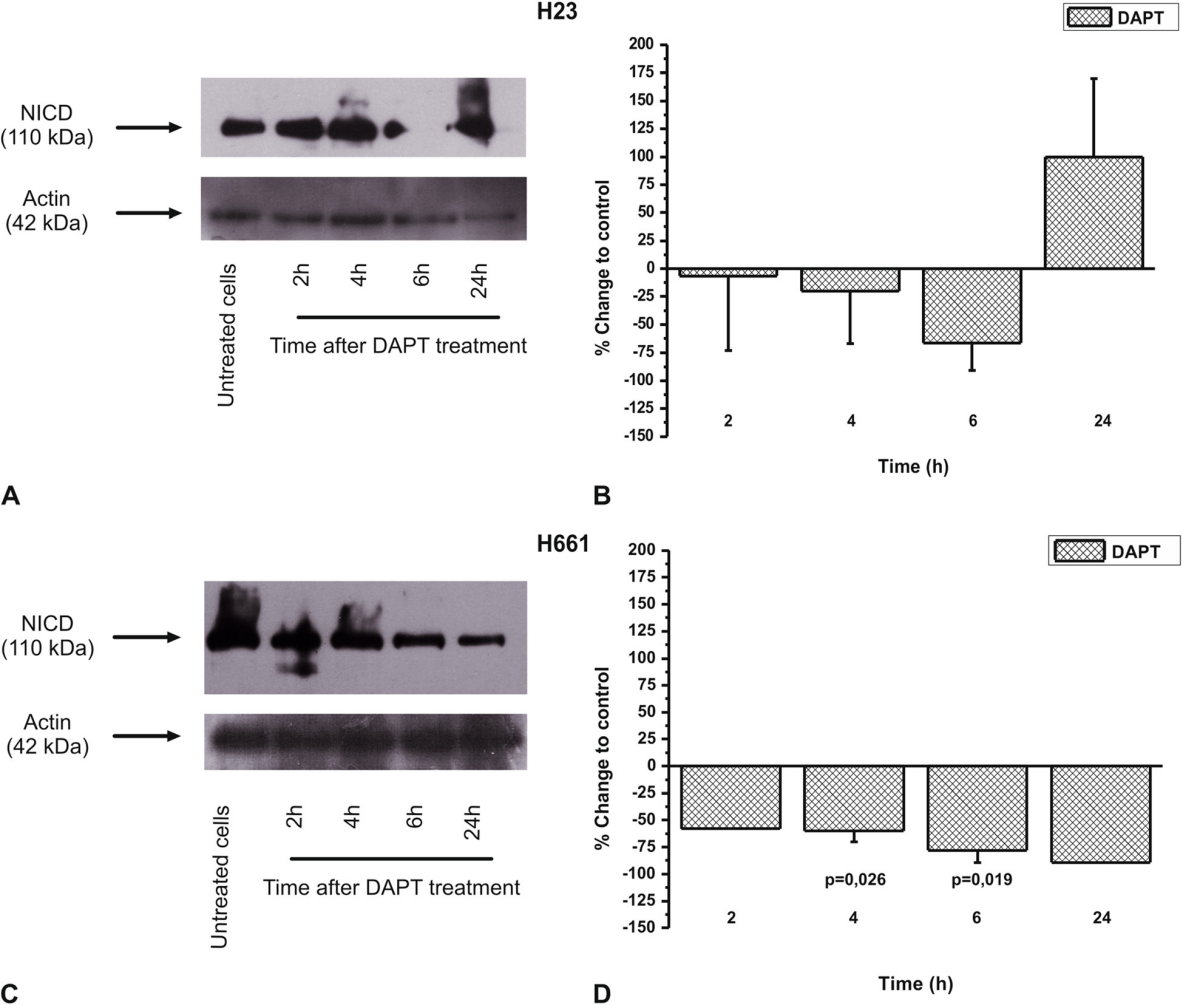
The DAPT effect on NICD protein levels in **a**) H23 and **c**) H661 cells in a time dependent manner. The images are representative of three independent experiments. Western blots were semi-quantified using appropriate software. The results in **b**) and **d**) are expressed as % change ± SEM compared to untreated cells from at least three independent experiments

**Fig. 4:**
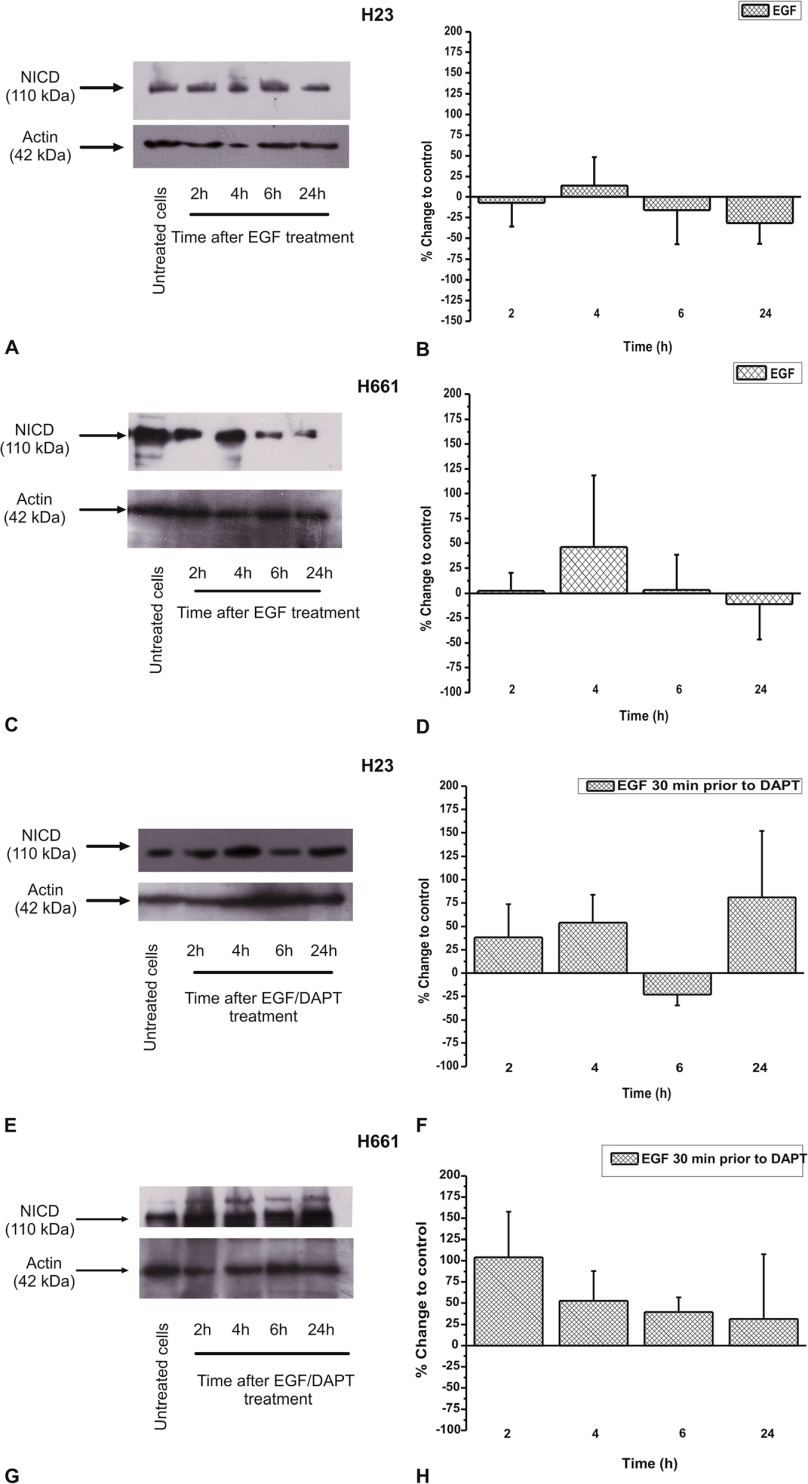
The EGF effect on NICD protein levels, alone in **a**) H23 and **c**) H661 cells or prior to DAPT in **e**) H23 and **g**) H661 cells in a time dependent manner. The images are representative of three independent experiments. Western blots were semi-quantified using appropriate software. The results in **b**), **d**), **f**) and **h**) are expressed as % change ± SEM compared to untreated cells from at least three independent experiments

In order to correlate the effect of DAPT on cell proliferation with inhibition of the Notch pathway, we checked the effect of DAPT on NICD protein levels. DAPT decreased NICD protein levels in both H23 and H661 cells, reaching a maximum effect at 6 h (Fig. 3). Interestingly, DAPT was more effective in H661 cells, in line with its effect on the number of cells. We next studied the effect of EGF alone or in combination with DAPT. EGF alone did not cause any statistical significant effect on NICD protein levels either in H23 (Fig. 4a and b) or in H661 (Fig. 4c and d) cells. When EGF was applied 30 min prior to DAPT, it prevented NICD decrease caused by DAPT, in both H23 and H661 cells (Fig. 4e, f, g and h), in line with the corresponding effects on the number of cells. In case of H23 cells, this prevention was observed up to 4 h after cells’ treatment, while at 6 h there was an attenuation effect of EGF to DAPT-induced NICD protein decrease (Fig. 4e and f). In H661 cells there was a full prevention at all-time points tested (Fig. 4g and h).

**Fig. 5:**
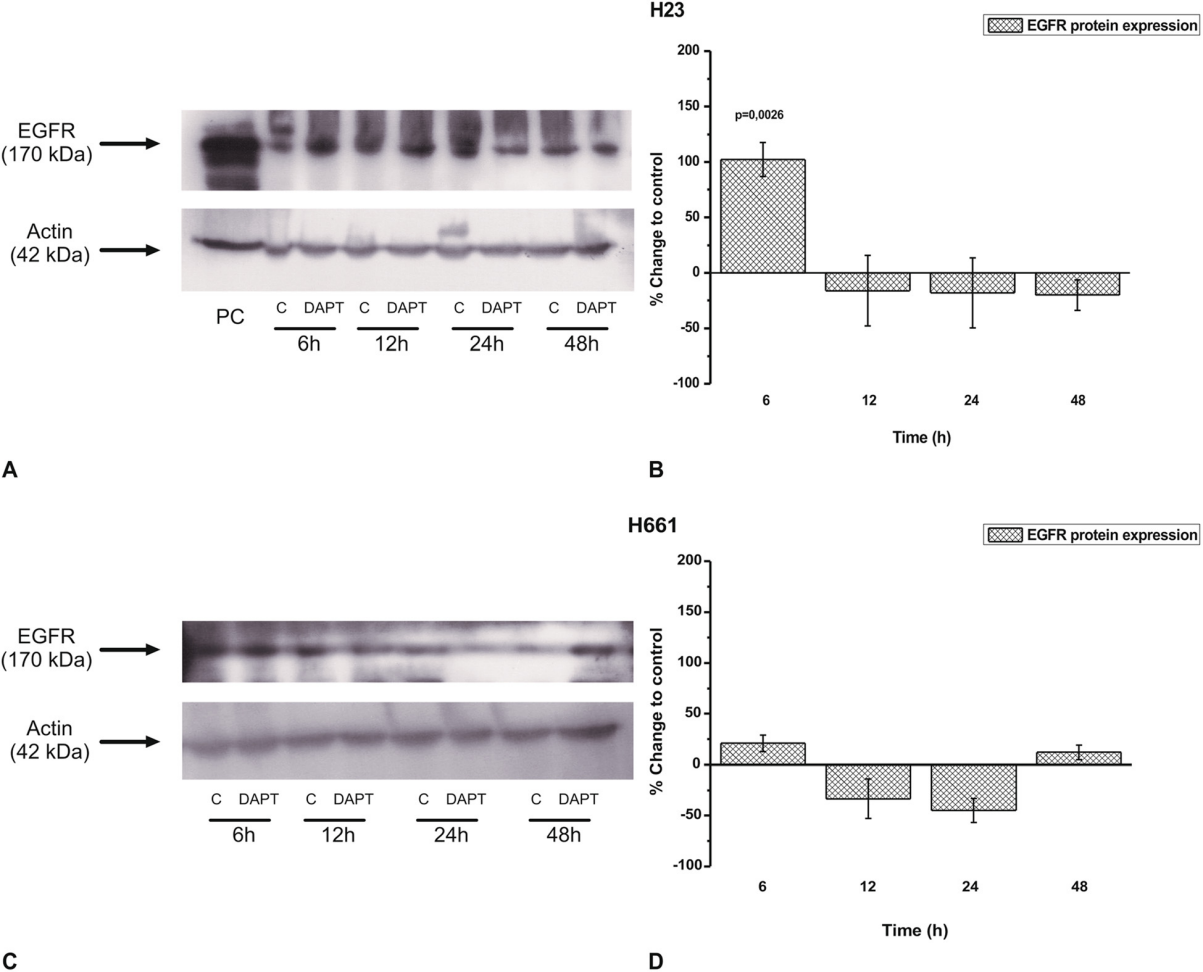
The effect of DAPT in EGFR protein levels in several time points in **a**) H23 and **c**) H661 cells. The images are representative of three independent experiments. Western blots were semi-quantified using appropriate software. The results in **b**) and **d**) are expressed as % change ± SEM compared to untreated cells from at least three independent experiments

We then tested whether DAPT affected EGFR protein levels in lung cancer cells. DAPT increased the protein levels of EGFR in H23 (Fig. 5a) but not H661 cells (Fig. 5b).

### Notch inhibition in NSCLC cells’ death

**Fig. 6:**
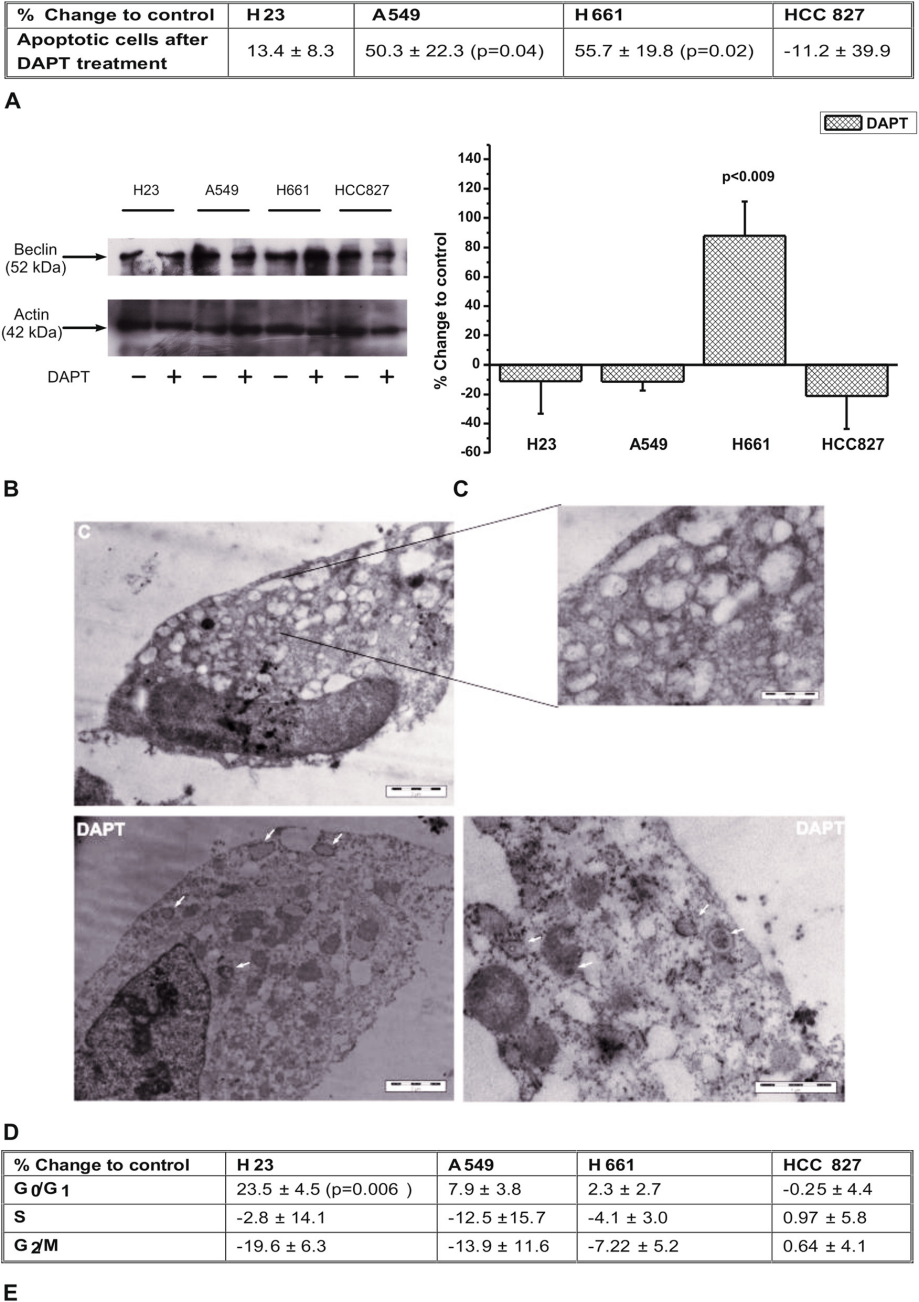
The effect of DAPT in NSCLC cell death. **a** Apoptosis is expressed as the % change ± SEM compared to untreated cells from at least three independent experiments. **b** Autophagy is estimated according to Beclin-1 protein levels. The image is a representative of three independent experiments. **c** The images from western blot analysis were semi-quantified using appropriate software. **d** Autophagosomes were detected in H661 cells after their treatment with DAPT under observation of samples using TEM. **e** Cell cycle arrest is expressed as % change ± SEM compared to untreated cells from at least three independent experiments

We estimated whether the reduction in cell number after DAPT application in lung cancer cells was due to stimulation of any of the two types of programmed cell death, apoptosis and autophagy. DAPT did not affect apoptosis in H23 and HCC827 cells, but increased cell apoptosis in A549 and H661 cells in a statistically significant manner (Fig. 6a and Additional file 2: Figure S1). Regarding autophagy, we found that DAPT increased the protein levels of Beclin-1, an indicator of autophagy [28, 29], only in H661 cells. This increase was 88 % ± 23 and was observed 24 h after DAPT addition to cells (Fig. 6b and c). Indeed, autophagosomes were detected in H661 cells 24 h after their treatment with DAPT, using TEM (Fig. 6d). Although the untreated cells appeared to have granular cytoplasm, no sign of autophagy was detected compared to cells after treatment with DAPT.

We then studied the effect of DAPT in cell necrosis and no significant differences were found in any cell line tested (data not shown).

Finally, we tested whether DAPT affected cell cycle distribution 24 h after DAPT application to cells and we found no effect (data not shown). However, DAPT increased to a small extent the percentage of H23 cells in G_0_/G_1_ phase at 48 h. A slight increase was also found in A549 cells, without being statistically significant. No significant effect was observed in H661 and HCC827 cells (Fig. 6e and Additional file 3: Figure S2).

## Discussion

The EGFR pathway plays a vital role in the pathogenesis and progression of NSCLC. Although the contribution of EGFR signalling in lung cancer development is well established, the importance of Notch pathway in lung cancer is under investigation. The existing data for the role of Notch in lung cancer are conflicting and not limited between NSCLC and SCLC groups, but differences are also demonstrated among NSCLC groups, as well as among the members of Notch family. Chen et al*.* reported that Notch 1 was down-regulated in NSCLC cell lines, while constitutive expression of active Notch 1 in NSCLC cells caused cell death depending on oxygen concentration [30]. Another study showed that Notch 3 is active in NSCLC and treatment of cells with a γ-secretase inhibitor caused a cell proliferation reduction and increase in apoptosis [17]. Yin et al*.* suggested that the controversial effects of Notch signaling are highly context-dependent [31]. In addition, it has been found that Notch effect might be dose-dependent in mammary epithelial MCF-10A cells, whereas high Notch activity caused inhibition of cell proliferation and low Notch activity stimulated a strong hyperproliferative response [32]. All these conflicting data reveal a significant but complicated role of Notch in cancer development and progression.

In the current study, we selected four NSCLC cell lines expressing different levels of NICD and EGFR protein levels. We found that the cell lines exhibited different response to the γ-secretase inhibitor DAPT and surprisingly, this behavior seems to be related to EGFR status. DAPT was effective in proliferation of cells expressing wtEGFR, while it did not affect HCC827 cells expressing mtEGFR. In addition, differences were observed among the cells with wtEGFR. We found that although H23 and A549 cells exerted a similar small response to DAPT regarding cell proliferation, the decrease in cell number was possibly due to cell cycle arrest for H23 cells and increase in apoptosis for A549 cells. In H661 cells that were more sensitive to DAPT, the decrease in cell number was due to an increase of both apoptosis and autophagy. Our results verify that the impact of Notch inhibition may vary depending on cell context, since different types of cell death occurred in different cell lines. Although in the literature both cell cycle arrest [33, 34] and apoptosis stimulation [17, 35] have been described to be induced in cancer cells by Notch inhibition, there is no previous evidence that Notch inhibition triggers autophagy in cancer cells. However, it is known that apoptosis and autophagy are two mechanisms of programmed cell death that may co-exist and act synergistically [36]. A link between Notch pathway and autophagy was presented in a recent paper where the authors observed that loss of autophagy leads to precocious Notch activation during Drosophila oogenesis [37]. We might assume that H661 cells were more sensitive to DAPT because of the dual induction of apoptosis and autophagy compared with H23 and A549 cells, where only one type of cell death was activated. The sensitivity of H661 cells to DAPT might be correlated with the EGFR protein levels, since H661 cells expressed the lowest EGFR levels compared with H23 and A549 cells. The low EGFR protein levels may render H661 cells more sensitive to EGFR-independent signaling pathways regarding cell proliferation. This hypothesis could be supported by the failure of EGF alone to stimulate H661 cell proliferation. Although, EGF failed to stimulate A549 cell proliferation too, this may be explained by the existence of Kras mutation in these cells [38].

Since our data indicated that the effect of γ-secretase inhibitors might be affected by the EGFR status, the three cell lines expressing wtEGFR were stimulated with EGF prior to DAPT addition. The stimulation of all cells with EGF fully prevented the inhibition of cell proliferation by DAPT. This is in agreement with the lack of effect of DAPT in HCC827 cells bearing active mtEGFR [26, 27]. Although our data are not conclusive, this is the first indication in the literature that EGFR activation may affect Notch signalling in NSCLC cells. A similar effect regarding NICD protein levels was observed when cells were treated with EGF prior to DAPT application. EGF attenuated the reduction of NICD levels caused by DAPT. Nevertheless, since there is no direct evidence for the impact of EGF to NICD protein, we might assume that ERK1/2 phosphorylation induced by EGF activates Notch pathway [39]. Regarding a bidirectional effect between Notch and EGFR pathway, to our knowledge, there is evidence from breast cancer cells where Notch over-expression caused EGFR up-regulation [19]. A similar effect has been described in gliomas through p53 regulation [40]. Αn opposite effect has been described in breast cancer cells where HER-2 inhibition using transtuzumab caused increased Notch 1 activity [41]. In this study, inhibition of Notch activation by DAPT increased EGFR protein levels in H23 cells without affecting the EGFR levels in H661 cells. We might speculate that this increase offered a relative resistance in H23 cells compared to H661 cells in cell proliferation after DAPT treatment.

This study presents indications that EGFR and Notch signalling pathways crosstalk in human lung cancer cell lines. In more recent research, it has been shown that in hypoxia, ADAM12 could be the linker between the two pathways since it mediates the release of heparin-binding EGF-like growth factor after Notch activation that leads to EGFR activation and cell invasion promotion. These experiments performed in head and neck, lung and pancreatic cancer cells [42].

## Conclusions

In conclusion, the interaction of Notch with EGFR revealed from our data might imply that a dual inhibition of these pathways might be promising in NSCLC cells that express high EGFR protein levels. This indicates an attractive new avenue of combination approaches for cancer therapy that may enhance the potency of EGFR inhibitory agents on tumours.

## Additional files

Additional file 1: Table S1. Classification of cells according to ATCC-LGC Promochem instructions (https://www.lgcstandards-atcc.org/), regarding tissue type of origin and EGFR mutation status. (PDF 26 kb) Additional file 2: Figure S1. Representative plots for apoptosis and necrosis detection in H23, A549, H661 and HCC827 cells as described in “Materials and Methods” section. Control: untreated cells and DAPT: cells treated with DAPT. Four populations are distinguished: the viable (An-/PI-) [region 3], the early apoptotic (An+/PI-) [region 4], the late apoptotic (An+/PI+) [region 2] and the necrotic (An-/PI+) [region 1] cells. (PDF 399 kb) Additional file 3: Figure S2. Representative plots for cell cycle analysis in H23, A549, H661 and HCC827 cells as described in “Materials and Methods” section. Control: untreated cells, DAPT: cells treated with DAPT. The G0/G1, S and G2/M phases are indicated with blue, red and green colour, respectively. (PDF 113 kb)
