# Supplementary material for: Epidermal growth factor receptor status and Notch inhibition in non-small cell lung cancer cells
Source: J Biomed Sci. 2015 Oct 24;22:98. doi: 10.1186/s12929-015-0196-1 (PMC4619334; doi:10.1186/s12929-015-0196-1)
Supplement: Additional file 1: Table S1. — Classification of cells according to ATCC-LGC Promochem instructions (https://www.lgcstandards-atcc.org/), regarding tissue type of origin and EGFR mutation status. (PDF 26 kb) [file 12929_2015_196_MOESM1_ESM.pdf]

Additional file 1

Table 1: Classification of cells according to ATCC-LGC Promochem instructions [24], regarding tissue type of origin and EGFR mutation status.

NSCLC cell lines	***Tissue type***	***EGFR status***
H23	Adenocarcinoma	wt
A549	Squamous cell carcinoma	wt
H661	Large cell carcinoma	wt
HCC827	Adenocarcinoma	mt

wt: wild type, mt: mutated
