# Supplementary material for: Epidermal growth factor receptor status and Notch inhibition in non-small cell lung cancer cells
Source: J Biomed Sci. 2015 Oct 24;22:98. doi: 10.1186/s12929-015-0196-1 (PMC4619334; doi:10.1186/s12929-015-0196-1)
Supplement: Additional file 2: Figure S1. — Representative plots for apoptosis and necrosis detection in H23, A549, H661 and HCC827 cells as described in “Materials and Methods” section. Control: untreated cells and DAPT: cells treated with DAPT. Four populations are distinguished: the viable (An-/PI-) [region 3], the early apoptotic (An+/PI-) [region 4], the late apoptotic (An+/PI+) [region 2] and the necrotic (An-/PI+) [region 1] cells. (PDF 399 kb) [file 12929_2015_196_MOESM2_ESM.pdf]

Additional file 2:

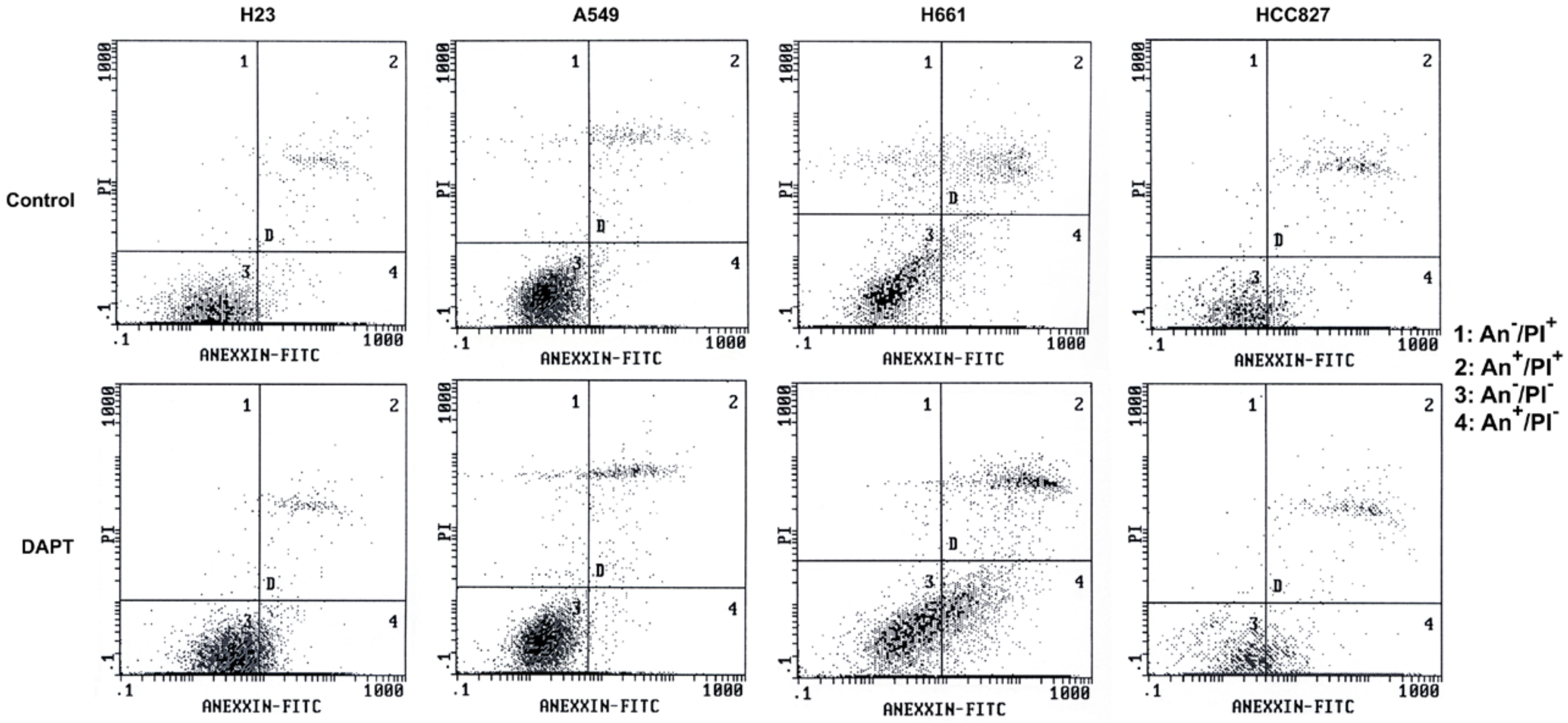

Figure 1: Representative plots for apoptosis and necrosis detection in H23, A549, H661 and HCC827 cells as described in "Materials and Methods" section. Control: untreated cells and DAPT: cells treated with DAPT. Four populations are distinguished: the viable (An^-/PI^-) [region 3], the early apoptotic (An^+/PI^-) [region 4], the late apoptotic (An^+/PI^+) [region 2] and the necrotic (An^-/PI^+) [region 1] cells.
