# Supplementary material for: Epidermal growth factor receptor status and Notch inhibition in non-small cell lung cancer cells
Source: J Biomed Sci. 2015 Oct 24;22:98. doi: 10.1186/s12929-015-0196-1 (PMC4619334; doi:10.1186/s12929-015-0196-1)
Supplement: Additional file 3: Figure S2. — Representative plots for cell cycle analysis in H23, A549, H661 and HCC827 cells as described in “Materials and Methods” section. Control: untreated cells, DAPT: cells treated with DAPT. The G0/G1, S and G2/M phases are indicated with blue, red and green colour, respectively. (PDF 113 kb) [file 12929_2015_196_MOESM3_ESM.pdf]

Additional file 3:

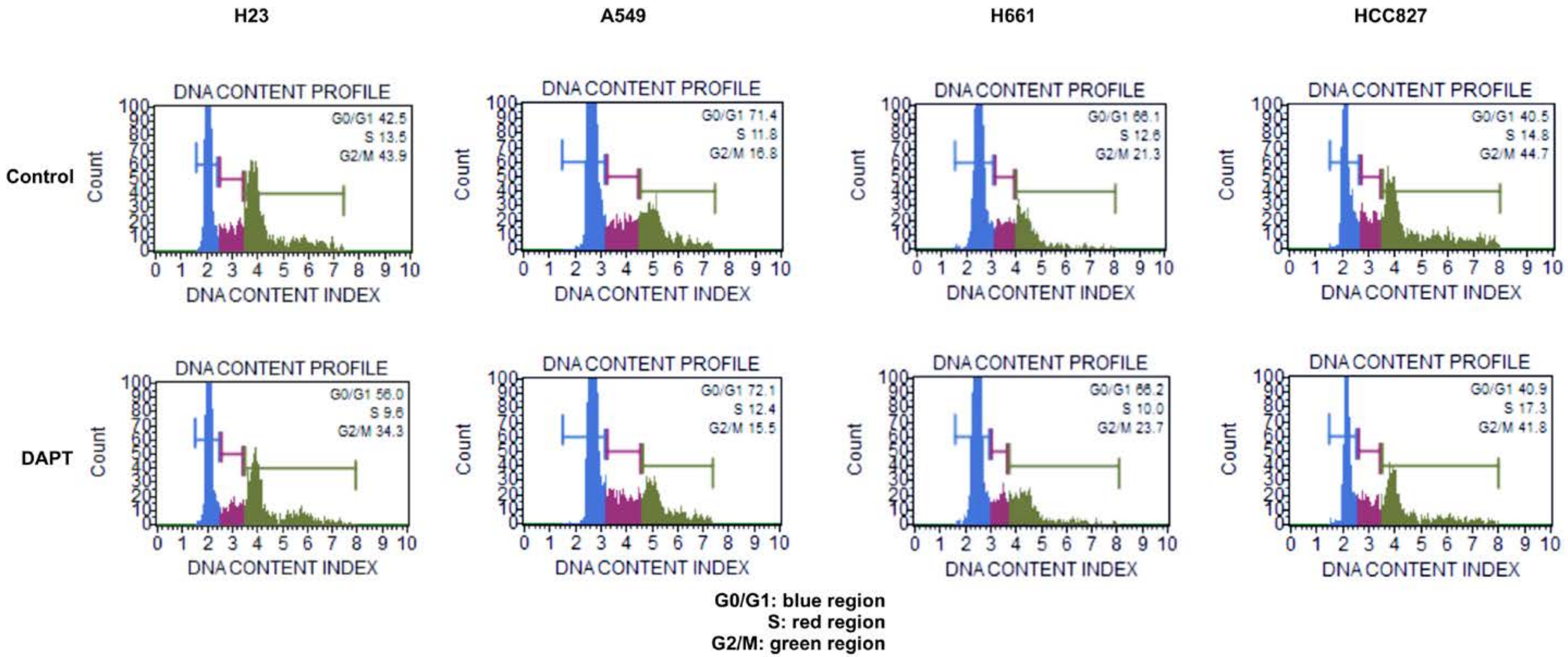

Figure 2: Representative plots for cell cycle analysis in H23, A549, H661 and HCC827 cells as described in "Materials and Methods" section. Control: untreated cells, DAPT: cells treated with DAPT. The G0/G1, S and G2/M phases are indicated with blue, red and green colour, respectively.
